# Methadone switching for refractory cancer pain

**DOI:** 10.1186/s12904-022-01076-2

**Published:** 2022-11-02

**Authors:** Haiying Ding, Yu Song, Wenxiu Xin, Jiao Sun, Like Zhong, Qinfei Zhou, Chaoneng He, Liyan Gong, Luo Fang

**Affiliations:** 1 Department of Pharmacy, The Cancer Hospital of the University of Chinese Academy of Sciences (Zhejiang Cancer Hospital) , Institute of Basic Medicine and Cancer (IBMC), Chinese Academy of Sciences, Hangzhou, China; 2 Zhejiang Key Laboratory of Prevention, Diagnosis and Therapy of Upper Gastrointestinal Cancer, Zhejiang Cancer Hospital, 310022 Hangzhou, China; 3 Department of Rare Cancer & Head and Neck Medical Oncology, The Cancer Hospital of the University of Chinese Academy of Sciences (Zhejiang Cancer Hospital), Institute of Basic Medicine and Cancer (IBMC), Chinese Academy of Sciences, Hangzhou, China

**Keywords:** Methadone, Refractory cancer pain, Efficacy, Safety, Cost, Opioids switching

## Abstract

**Background:**

Methadone is commonly considered an alternative opioid treatment for refractory cancer pain. This study aims to investigate the efficacy, safety, and cost of methadone in the treatment of refractory cancer pain.

**Methods:**

A retrospective study was conducted in patients who used methadone for refractory cancer pain from April 2016 to December 2020 at a cancer specialized hospital. Pain control, evaluated via pain score and breakthrough pain frequency, and adverse events of methadone were compared with analgesic regimens prior to methadone administration. The factors potentially affecting the switching outcome were analyzed via multivariate analysis. Moreover, the cost of pain control was estimated.

**Results:**

Ninety patients received methadone for poor pain control (74.4%), intolerable adverse events (10.0%), or both (15.6%) after prior opioid treatments. Sixty-four patients (71.1%) were successfully switched to methadone with median pain score significantly decreased from 4.0 to 2.0 (p < 0.001) and median daily frequency of breakthrough pain from 3.0 to 0.0 (p < 0.001) at a maintained median conversion ratio of 6.3 [interquartile range (IQR): 4.0–10.0] to prior opioid treatment. Similar adverse event profiles of constipation, nausea, vomiting, and dizziness were observed between methadone and prior opioid regimens. The median daily cost of analgesic regimens was significantly reduced from $19.5 (IQR: 12.3–46.2) to $10.8 (IQR: 7.1–18.7) (p < 0.01) after switching to methadone. The 3-day switch method significantly improved the rate of successful switching compared with the stop and go method (odds ratio = 3.37, 95% CI: 1.30–8.76, p = 0.013).

**Conclusion:**

Methadone is an effective, safe, and cost-saving treatment for patients with refractory cancer pain.

## Background

Pain is one of the most common and unbearable symptoms experienced by cancer patients; it seriously impairs their quality of life [1, 2]. Approximately 53% of cancer patients, and 60–70% of advanced cancer patients experience pain, more than one-third of which is moderate-to-severe [3]. Opioids, including morphine, oxycodone, and fentanyl, are cornerstones for the treatment of moderate-to-severe cancer pain [4]. However, 10–20% of patients who are prescribed opioids still experience inadequate pain relief and/or intolerable adverse reactions, which can be defined as refractory pain [5–7]. Methadone is commonly considered an alternative opioid treatment for refractory cancer pain [8–11].

Methadone is a synthetic µ-opioid receptor agonist that has a stronger affinity for δ opioid receptors than morphine [12]. Methadone is also an antagonist of the N-methyl-D-aspartic acid (NMDA) receptor and inhibits the reuptake of serotonin and norepinephrine [13, 14]. Given the advantages of methadone, including long-lasting analgesia, high oral bioavailability, good safety in patients with renal dysfunction, and low cost, its application in cancer pain treatment has attracted great attention [15]. Furthermore, the characteristics of extra-opioid analgesic action and anti-hyperalgesic properties make methadone a promising agent for the treatment of opioid resistance, central sensitization, and opioid-induced hyperalgesia [10, 16].

However, the application of methadone in cancer pain treatment remains challenging due to its complicated and inconsistent conversion ratio from pre-switching opioid dose to methadone [17]. Additionally, adverse methadone-related cardiac events are of concern, especially cardiotoxicity of prolonged corrected QT interval (QTc) and torsade de pointes (TdP) [18, 19]. Clinical data on methadone for cancer pain management are still limited, especially for Asian patients [8, 9, 17, 20]. Therefore, the present study aimed to evaluate the efficacy, tolerance, and economy of opioid switching to methadone in the management of refractory cancer pain.

### Methods

A retrospective study was conducted in patients who used methadone for cancer pain in Zhejiang Cancer Hospital from April 2016 to December 2020.

Patients were included if they met the following inclusion criteria: (1) they had methadone prescribed for compromised pain control and/or intolerable opioid-related adverse reactions to opioids, including long-acting morphine and oxycodone, transdermal fentanyl, and patient-controlled analgesia; (2) they had available data on pain control, adverse events, and cost of both methadone and opioid regimens prior to methadone administration. This study conformed with the principles of the Declaration of Helsinki and was approved by the institutional ethics board of the Zhejiang Cancer Hospital (No. IRB-2022-86).

Data on patient demographics, cancer diagnosis, disease stage, bone metastasis, pain type, and Karnofsky score were collected. Details regarding previously prescribed opioids, reason for methadone prescription and discontinuation, methods of methadone switching, initial and maintenance doses of methadone, and pain control and adverse events of both methadone and prior opioids were also recorded. Pre- and post-switching analgesia efficacy was assessed and compared using the pain intensity score and daily frequency of breakthrough pain (BTP). Pain intensity scores were measured using the numeric rating scale (NRS) (ranging from 0 to 10, with 0 = no pain and 10 = worst pain imaginable). Successful switching of methadone was defined as adequate pain relief (pain intensity score ≤ 3 requiring no more than three supplemental doses for breakthrough pain per 24 h) with no occurrence of intolerable adverse events. Adverse events were assessed using the National Cancer Institute Common Terminology Criteria for Adverse Events (version 5.0) [21].

The oral morphine equivalent daily dose (OMEDD) of long-acting opioids and opioids for patient-controlled analgesia (PCA) was calculated according to the recommendation of opioid equivalences and relative potency of the National Comprehensive Cancer Network (NCCN) clinical practice guidelines for cancer pain [22]. The conversion ratio from opioids to methadone was calculated by dividing the OMEDD of prior opioids by the dosage of methadone.

Costs of analgesic regimens were calculated based on the costs of disposable infusion devices for electric infusion pumps and drugs for pain control: oxycodone hydrochloride prolonged-release tablets, fentanyl transdermal patches, methadone hydrochloride tablets, sufentanil citrate injection, hydromorphone hydrochloride injection, morphine hydrochloride injection, celecoxib, etoricoxib, and adjuvant analgesics such as gabapentin and pregabalin. Values/costs in Chinese Yuan were converted to US dollars (1 USD = 6.3715 CNY).

The Wilcoxon signed rank test was used to compare efficacy and cost before and after methadone switching, and the chi-squared test was used to analyze differences in the incidence of adverse reactions between methadone and other opioids. Binary logistic regression was conducted to evaluate the relationship between successful switching and factors including types of pain, reasons for switching, methods of switching, NRS before switching, BTP before switching, opioids prior to switching, and OMEDD of prior opioids. Odds ratios (ORs), including 95% confidence intervals, were calculated. Subsequently, multivariate analysis was performed to evaluate independent predictors of successful switching using the backward stepwise elimination method. Furthermore, subgroup analysis of subpopulations with compromised pain control was performed. Statistical analyses were performed using SPSS Statistics 23.0, and statistical significance was set at p < 0.05.

## Results

### Background characteristics

A total of 90 patients, mostly with neuropathic pain (60.0%), were included (Table 1). The primary diseases were lung cancer (38.9%, n = 35), colorectal cancer (12.2%, n = 11), and cervical cancer (7.8%, n = 7). All patients were in the advanced stage, and the majority (58.9%) had accompanying bone metastasis. For patients who experienced unsuccessful switching compared with all patients, there were more females (53.8% vs. 40.0%), and the proportion of patients with a Karnofsky score of 50 seemed higher (26.9% vs. 17.8%). Besides, more patients present with nociceptive pain alone in the unsuccessful group (53.8% vs. 40.0%).

**Table 1:** Patient demographics, N (%)

Demographics	All patients N = 90	Unsuccessful switching N = 26
Gender		
Male	54 (60.0)	12 (46.2)
Female	36 (40.0)	14 (53.8)
Age (years), mean (SD)	53.4 (11.7)	56.7 (12.0)
≥ 60	33 (36.7)	13 (50.0)
Primary diagnosis		
Lung cancer	35 (38.9)	11 (42.3)
Colorectal cancer	11 (12.2)	3 (11.5)
Cervical cancer	7 (7.8)	3 (11.5)
Gastric cancer	5 (5.6)	0 (0.0)
Breast cancer	4 (4.4)	1 (3.8)
Esophageal cancer	4 (4.4)	1 (3.8)
Others	24 (26.7)	7 (26.9)
Disease stage		
Stage III	2 (2.2)	1 (3.8)
Stage IV	88 (97.8)	25 (96.2)
Karnofsky score		
50	16 (17.8)	7 (26.9)
60	50 (55.6)	14 (53.8)
70	21 (23.3)	3 (11.5)
80	3 (3.3)	2 (7.7)
Bone metastasis		
Yes	53 (58.9)	16 (61.5)
Pain type		
Nociceptive pain	36 (40.0)	14 (53.8)
Neuropathic pain	20 (22.2)	5 (19.2)
Mixed pain	34 (37.8)	7 (26.9)
QTc, mean (SD)	0.35 (0.03)	0.35 (0.03)

### Methadone switching

Prior to methadone treatment, patients were prescribed extended-release oxycodone (61.1%), transdermal fentanyl, PCA, long-acting opioids combined with PCA, or extended-release oxycodone plus transdermal fentanyl (Table 2). Most patients (74.4%) switched to methadone treatment because of poor pain control. The initial and maintenance conversion ratios were 7.3 with an interquartile range (IQR) of 5.0–11.1 and 6.3 (IQR 4.0–10.0). Finally, 64 patients (71.1%) achieved successful methadone switching. There were 17 (18.9%) and 9 (10.0%) patients who failed to switch because of unsatisfactory pain control and intolerable adverse events, including nausea and vomiting (n = 6), respiratory depression (n = 1), arrhythmia (n = 1), and delirium (n = 1), respectively. Fifty (55.6%) and forty (44.4%) patients were switched to methadone via the 3-day switch (3DS) and stop and go (SAG) strategies, respectively. In the case of SAG, the current opioid is immediately substituted with methadone [23, 24], while for 3DS, the dose of the current opioid is substituted stepwise with methadone over three days [25, 26]. Univariate analysis revealed that switching via the 3DS strategy significantly improved the rate of successful switching (OR = 3.37, 95% CI: 1.30–8.76, p = 0.013) compared with switching via the SAG strategy. Moreover, gender was seemingly related to switching success (male vs. female: OR = 2.22, 95% CI: 0.88–5.56, p = 0.091). As revealed by the multivariate analysis, 3DS-switching independently predicted successful switching compared to switching via SAG (OR = 3.24, 95% CI: 1.19–8.85, p = 0.022). Compared to patients using the SAG method, fewer discontinued methadone (4% vs. 22.5%, p = 0.008) and high successful switching (82.0% vs. 57.5%, p = 0.011) of those using the 3DS method was investigated.

**Table 2:** Comparison of characteristics of successful and unsuccessful methadone switching, N (%)

	All patients	Successful switching	Unsuccessful switching
Total	N = 90	64 (71.1)	26 (28.9)
Previously prescribed opioids			
Extended-release oxycodone	55 (61.1)	41 (74.5)	14 (15.5)
Fentanyl transdermal patch	9 (10.0)	5 (55.5)	4 (44.5)
PCA^*^	7 (7.8)	6 (85.7)	1 (14.3)
Extended-release oxycodone or transdermal fentanyl combined with PCA	18 (20.0)	11 (61.1)	7 (38.9)
Extended-release oxycodone combined with fentanyl transdermal patch	1 (1.1)	1 (100.0)	0 (0.0)
Reasons for switching			
Poor pain control	67 (74.4)	51 (76.1)	16 (23.9)
Intolerable adverse events	9 (10.0)	5 (55.6)	4 (44.5)
Both	14 (15.6)	8 (57.1)	6 (42.9)
Switching method			
3-day switch	50 (55.6)	41 (82.0)	9 (18.0)
Stop and go	40 (44.4)	23 (57.5)	17 (42.5)
OMEDD pre-switching			
< 300 mg	40 (44.4)	24 (60.0)	16 (40.0)
300–600 mg	32 (35.6)	26 (81.3)	6 (18.8)
> 600 mg	18 (20.0)	14 (77.8)	4 (22.2)
Conversion ratio			
Under calculated ratio	53 (58.9)	39 (73.5)	14 (26.4)
Matched calculated ratio	13 (14.4)	9 (69.2)	4 (30.8)
Over calculated ratio	24 (26.7)	16 (66.7)	8 (33.3)

Note: OMEDD Oral Morphine Equivalent Daily Dose; PCA Patient-Controlled Analgesia

A comparison of the actual initial conversion ratio from opioids to methadone and the recommended initial conversion ratio derived from guidelines [27] is shown in Table 3. The actual initial conversion ratios were slightly lower than recommended, implying that the initial methadone doses might have been higher than recommended. The initial conversion ratios were similar in the successful and unsuccessful switching groups (median: 7.3, IQR: 5.4–10.6 vs. median: 7.4, IQR: 4.0–14.2).

**Table 3:** Conversion ratio from opioids to methadone

OMEDD pre-switching (mg)	Recommended initial CR	All N (%)	Initial CR Median (IQR)	Successful switching						Failure switching				
				N	Initial CR Median (IQR)	Under calculated ratio	Matched calculated ratio	Over calculated ratio		N	Initial CR Median (IQR)	Less than calculated ratio	Equal to calculated ratio	More than calculated ratio
All	—	90	7.3 (5.0–11.1)	64	7.3 (5.4–10.6)	39 (60.9)	9 (14.1)	16 (25.0)		26	7.4 (4.0–14.2)	14 (53.8)	4 (15.4)	8 (30.8)
30–90	4:1	15 (16.7)	3.3 (3.0–5.0)	9	4.0 (3.2–5.5)	4 (44.4)	2 (22.2)	3 (33.3)		6	3.2 (2.8–4.5)	4 (66.7)	1 (16.7)	1 (16.7)
91–300	8:1	27 (30.0)	6.0 (5.0–8.0)	16	6.0 (5.3–8.0)	10 (62.5)	3 (18.8)	3 (18.8)		11	6.7 (4.7–8.3)	6 (54.5)	2 (18.2)	3 (27.3)
301–600	10:1	31 (34.4)	8.0 (6.4–11.5)	26	8.0 (6.3–10.1)	17 (65.4)	4 (15.4)	5 (19.2)		5	16.8 (8.0–21.6)	2 (40.0)	0 (0.0)	3 (60.0)
601–800	12:1	4 (4.4)	9.2 (6.2–19.1)	3	5.6, 10.4, 22.0	2 (66.7)	0 (0.0)	1 (33.3)		1	8.0	1 (100.0)	0 (0.0)	0 (0.0)
801–1000	15:1	3 (3.3)	6.4, 10.7, 10.0	3	6.4, 10.7, 10.0	3 (100.0)	0 (0.0)	0 (0.0)		0	—	—	—	—
> 1000	20:1	10 (11.1)	22.4 (13.9–49.2)	7	24.3 (14.7–65.2)	3 (42.9)	0 (0.0)	4 (57.1)		3	43.8, 20.4, 11.4	1 (33.3)	1 (33.3)	1 (33.3)

Note: OMEDD Oral Morphine Equivalent Daily Dose; CR Conversion Ratio; IQR Interquartile Range

### Pain control and cost

The median pain score of patients with successful switching was significantly decreased from 4.0 (IQR 2.0–5.0) to 2.0 (IQR 2.0–2.0, p < 0.001), and the median daily frequency of breakthrough pain was significantly reduced from 3.0 (IQR 0.0–5.0) to 0.0 (IQR 0.0–1.0, p < 0.001) by methadone. Moreover, switching to methadone significantly reduced the daily cost of cancer pain treatment by 45% from $19.5 (IQR 12.3–46.2) to $10.8 (IQR 7.1–18.7), p < 0.001.

### Adverse events

Constipation, nausea, vomiting, and dizziness were the most common adverse reactions for both methadone and prior opioids (Table 4). No significant difference in the incidence of adverse events was observed between methadone and other opioids, except for urinary retention with a significantly decreased risk with methadone treatment (0.0% vs. 4.4%, p = 0.043). Moreover, increased risks of delirium (7.8% vs. 3.3%, p = 0.193) and decreased risks of pruritus (0.0% vs. 2.9%, p = 0.155) were observed after switching to methadone, although no statistical difference was observed. Only one patient discontinued methadone due to severe arrhythmia.

**Table 4:** Adverse events pre- and post-switching to methadone, N (%)

Adverse events	Pre-switching	Post-switching	P value
Constipation	25 (27.8)	21 (23.3)	0.496
Nausea	23 (25.6)	21 (23.3)	0.343
Vomiting	15 (16.7)	19 (21.1)	0.447
Dizziness	13 (14.4)	14 (15.6)	0.835
Decreased appetite	12 (13.3)	8 (8.9)	0.343
Hidrosis	6 (6.7)	3 (3.3)	0.347
Somnolence	4 (4.4)	3 (3.3)	0.700
Urinary retention	4 (4.4)	0 (0.0)	0.043
Delirium	3 (3.3)	7 (7.8)	0.193
Pruritus	2 (2.2)	0 (0.0)	0.155
Arrhythmia	0 (0.0)	1 (1.1)	0.316
Respiratory depression	0 (0.0)	1 (1.1)	0.316
Prostation	0 (0.0)	1 (1.1)	0.316
Blurred vision	0 (0.0)	1 (1.1)	0.316
Total	51 (56.7)	57 (63.3)	0.447

## Discussion

Approximately 10–20% of patients experience refractory cancer pain that is poorly responsive to commonly used opioids such as morphine, oxycodone, and fentanyl, or who have dose-limiting intolerable adverse effects [5, 6, 9]. Methadone is considered an attractive alternative in these situations [8, 9, 11]. This study evaluated the effect of methadone on refractory cancer pain in patients in China. 71% of patients were successfully switched to methadone, allowing for significantly improved pain control and tolerable adverse events. The adverse events for methadone were similar to those of prior opioids. The results indicated that switching from opioids to methadone could be a viable alternative in the treatment of refractory cancer pain.

Approximately three to four patients achieved good pain control after switching to methadone, which is similar to the previously reported conversion success rates of 77.4 [28], 80.0 [29], and 87.5% [30]. Methadone is structurally distinct from the opium-like alkaloids of most opioids, and it may be effective in patients who are resistant to opioids with opium-derived alkaloids [31]. First, methadone exhibits a higher affinity for δ receptors, which may partially account for its reduced cross-tolerance to other opioids [12]. Moreover, unlike other opioids, methadone is an NMDA receptor antagonist. NMDA plays a vital role in the development of hyperalgesia and opioid resistance [31]. Further, methadone inhibits serotonin and norepinephrine reuptake, which mediates the downward modulation of pain [31].

The conversion ratio from prior opioids to methadone is crucial for successful switching [19]. There are various recommendations for calculation of the methadone conversion ratio [17, 18, 27, 32]. At our hospital, doctors managed methadone switching mainly according to NCCN clinical practice guidelines for adult cancer pain (2019 v1) [27], which was derived from Ayonrinde and Bridge’s suggestion [32]. On March 15, 2019, the NCCN guideline (2019 v2) updated the recommendations for the methadone conversion ratio according to the Hospice and Palliative Medicine White Paper [18], the White Paper considered to be the easiest to implement, whereas clinicians in our hospital still performed methadone switching based on the old recommendations in the NCCN guideline (2019 v1). In consideration of safety, the recommended initial methadone dose was much lower according to the White Paper than the NCCN guideline (2019 v1), especially for patients with high doses of opioids. Therefore, patients may need more breakthrough analgesics and longer methadone titration based on the White Paper recommendation to achieve an optimized maintenance dosage. Patients would achieve good pain control more quickly using the more precise conversion ratio in the NCCN guideline (2019 v1). Moreover, our study indicated that patients who switched to methadone following the NCCN guideline (2019 v1) did not show significantly higher adverse effects compared with prior opioids (63.3% vs. 56.7%, p = 0.447).

Our results indicated that methadone-related adverse events were similar to those for opioids prior to switching. The occurrence of urinary retention decreased after patients switched to methadone. Conversely, methadone-related delirium was slightly increased. Numerous studies have reported methadone-induced delirium [33–36]. The incidence of delirium increased from 16 to 34% with the addition of low-dose methadone to high-dose opioids in terminally ill cancer patients [36]. Moreover, 4% of patients were discontinued due to delirium with first-line methadone for cancer pain [35]. However, the mechanisms by which methadone may increase the occurrence of delirium remain unclear. Additionally, some special adverse events of methadone, such as arrhythmia, prostation, and blurred vision, were observed. Methadone is widely acknowledged for its cardiac toxicity, such as prolongation of the QTc and occurrence of TdP [37]. Therefore, in this study, the risk of cardiotoxicity was assessed using baseline and follow-up electrocardiography [19, 22] before methadone switching and with methadone treatment, and methadone treatment was avoided in high-risk patients. As a result, only one patient developed severe arrhythmia that led to methadone withdrawal.

SAG and 3DS are two commonly used strategies for switching to methadone [7]. Our study indicated that the 3DS method achieved higher successful switching than the SAG method (83.3% vs. 56.8%). This was consistent with the results of previous studies [25, 26]. A randomized study revealed that the SAG approach was associated with compromised pain control, a higher number of dropouts, and more serious adverse events than the 3DS approach [25]. A subsequent study showed that the SAG strategy had no observed clinical benefit and a higher dropout rate [26]. Our study demonstrated that fewer patients using 3DS discontinued methadone treatment compared with those using SAG (4% vs. 22.5%, p = 0.008), which suggested that 3DS might be better tolerated than SAG. 3DS may avoid methadone accumulation and toxicity, especially in patients receiving high doses [25, 26].

This study had some limitations. First, given its retrospective nature, we were unable to obtain an accurate incidence of methadone-associated adverse effects. Second, this study was limited by its small size and single study site. A multicenter, prospective randomized study is necessary to confirm the efficacy and safety of methadone for refractory cancer pain.

## Conclusion

For patients with refractory cancer pain who are poorly responsive or intolerable to commonly used opioids, opioid switching to methadone has a satisfactory analgesic effect, good safety, and significantly reduced cost. Methadone is an effective choice for patients with refractory cancer pain, although comprehensively evaluating and closely monitoring adverse reactions remains necessary.

## Data Availability

The datasets generated and/or analysed during the current study are not publicly available due to institutional regulations but are available from the corresponding author on reasonable request.

## Abbreviations

NMDA: N-methyl-D-aspartic acid.
QTc: corrected QT interval.
TdP: torsade de pointes.
BTP: breakthrough pain.
NRS: numeric rating scale.
OMEDD: oral morphine equivalent daily dose.
PCA: patient-controlled analgesia.
NCCN: National Comprehensive Cancer Network.
OR: odds ratio.
IQR: interquartile range.
3DS: 3-day switch.
SAG: stop and go.
CR: conversion ratio.
